# Nutritional Considerations for the Vegan Athlete

**DOI:** 10.1016/j.advnut.2023.04.012

**Published:** 2023-04-29

**Authors:** Sam West, Alistair J. Monteyne, Ino van der Heijden, Francis B. Stephens, Benjamin T. Wall

**Affiliations:** Public Health and Sport Sciences, Faculty of Health and Life Sciences, University of Exeter, Exeter, United Kingdom

**Keywords:** vegan, athlete, sport, plant-based, adaptation, performance, skeletal muscle, dietary protein

## Abstract

Accepting a continued rise in the prevalence of vegan-type diets in the general population is also likely to occur in athletic populations, it is of importance to assess the potential impact on athletic performance, adaptation, and recovery. Nutritional consideration for the athlete requires optimization of energy, macronutrient, and micronutrient intakes, and potentially the judicious selection of dietary supplements, all specified to meet the individual athlete’s training and performance goals. The purpose of this review is to assess whether adopting a vegan diet is likely to impinge on such optimal nutrition and, where so, consider evidence based yet practical and pragmatic nutritional recommendations. Current evidence does not support that a vegan-type diet will enhance performance, adaptation, or recovery in athletes, but equally suggests that an athlete can follow a (more) vegan diet without detriment. A clear caveat, however, is that vegan diets consumed spontaneously may induce suboptimal intakes of key nutrients, most notably quantity and/or quality of dietary protein and specific micronutrients (eg, iron, calcium, vitamin B12, and vitamin D). As such, optimal vegan sports nutrition requires (more) careful consideration, evaluation, and planning. Individual/seasonal goals, training modalities, athlete type, and sensory/cultural/ethical preferences, among other factors, should all be considered when planning and adopting a vegan diet.

**Statement of Significance**

This article provides a comprehensive overview of the nutritional considerations for any athlete adopting a (more) vegan diet. The objective of this work is to highlight the key challenges faced by any athlete following a more plant-based diet, and where possible, provide nutritional and supplemental strategies to mitigate any issues.

## Introduction

Providing optimal nutrition for the athlete is a complex and challenging process. The breadth of consideration across athlete type, level, priorities (eg, training compared with competition, strength compared with endurance, weight gain compared with weight loss, etc), and personal characteristics (eg, age, sex, size, etc) all provide ever changing goalposts as to what constitutes ‘optimal nutrition.’ To constantly adapt for optimal nutrition requires the consideration of a wide array of foods. One challenge to this is the food preferences or dietary choices of the athlete. Although such restrictions can come in many forms (eg, cultural, religious, social, liking, pre-existing beliefs, non–sport-related priorities, etc), limiting the intake of animal-derived foods, at least to some degree, is an increasing priority. Table 1 displays the most common terms (which are rapidly increasing and evolving) used to describe different gradations of such diets [[1], [2]].

**TABLE 1:** Diet definitions

Type of Diet	Definition	Synonyms
Carnivore	Includes animal products only. Excludes vegetables, fruit, grains, legumes, nuts, seeds or starches.	Meat-eater.
Omnivore	Consumes a combination of plant and animal-based products.	
Macrobiotic^1^	Devoid of most flesh foods (might include fish). Excludes eggs, dairy, tropical fruits, processed sweeteners, and vegetables from the nightshade family (eg, potatoes, tomatoes, eggplant, and peppers).	
Flexitarian	Someone who actively tries to reduce animal product consumption. Encompasses a broad range of animal product consumption (some people may eat meat once a week, some people may have one meat free day a week).	Semi-vegetarian.
Pesco-vegetarian	Includes fish but excludes all animal flesh.	Pescetarian.
Pollo-vegetarian	Exclude red meat, fish and seafood. Includes poultry and fowl.	Pollotarian.
Vegetarian^2^	Devoid of flesh foods. May include egg or dairy products.	Veggie, herbivore.
Lacto-ovo-vegetarian^2^	Devoid of flesh foods. Includes eggs and dairy products.	
Lacto-vegetarian^2^	Devoid of flesh foods. Includes dairy products, but not egg products.	
Ovo-vegetarian^2^	Devoid of flesh foods. Includes egg products but not dairy.	
Fruitarian	Devoid of flesh foods. Includes fruit, seeds, nuts, and some vegetables.	
Vegan^2^	Devoid of flesh foods. Excludes all animal products (may include honey).	Plant-based, plant powered.
Raw Vegan^2^	Devoid of flesh foods. Based on vegetables, fruit, nuts and seeds, legumes and sprouted grains.	

1 Definition from [1].

2 Definitions from [2]

Veganism is of particular interest within sports nutrition due to being one of the more extreme forms of dietary restriction. Following a vegan diet (often extending to lifestyle choices beyond diet) involves eliminating all animal-based products, including meat, poultry, dairy, seafood, eggs, honey, gelatin, and rennet. A vegan, or ‘plant-based,’ diet is becoming increasingly popular in the general population [3], particularly in Western cultures [4]. The most frequently stated incentives are perceived health benefits [5,6] and ethical beliefs (eg, animal welfare concerns), although environmental concerns [7,8], social factors and sensory disgust [9] are also cited [9,10]. The prevalence of veganism in athlete populations is yet to be adequately quantified; however, it is reasonable to assume that veganism in sport will follow a similar trend to that of the general population. Of particular interest to athletes, in addition to the myriad reasons listed above, is emerging anecdotal reports that adopting a vegan diet might provide training and/or performance advantages. This is likely partially driven by increasing visibility and marketability of elite athletes following vegan diets, such as Venus Williams, Lewis Hamilton, Scott Jurek, David Haye and Fabian Delph, some of whom often purport athletic benefits to their dietary choice. Allied to these anecdotal claims, the popularity of high-profile documentaries (such as ‘The Game Changers’), and the discussion they generate, may further increase the interest in adopting a plant-based diet alongside training. Accepting this rising popularity, it is relevant to provide an evidence-based analysis regarding performance and/or training benefits. It is perhaps surprising that there are not more robust empirical reviews evaluating the effect of a vegan diet on training adaptation and performance outcomes. However, this becomes understandable when one considers the breadth that such an investigation would need to encompass, given the number of relevant variables, for example, the endpoint assessed (ie, performance, adaptation, recovery), the measures of that endpoint used (eg, time trial, gains in strength, glycogen resynthesis, etc), and the type of athlete (eg, endurance, power, team sport). Further, it is likely that each endpoint/measure/type of athlete is going to be affected differently by adopting a vegan diet. Therefore, drawing a set of definitive and quantitative conclusions for the effects of a vegan diet on an athlete’s lifestyle is challenging. This paper aims to provide a comprehensive narrative review of the theoretical factors that should be considered by any athlete (considering) following a vegan diet. As such, we address the contemporary and consequential question arising for all those with a vested interest in sports nutrition; ‘can an athlete adopt a vegan (type) diet and improve, or at least not impair, optimal athletic goals?’

### Vegan Sports Nutrition; *Defining Deficiency Compared with Suboptimal*

To consider the advantages or drawbacks of vegan nutrition within sports it is important to draw distinctions between nutritional *deficiencies* and *suboptimal* intakes. A nutritional deficiency refers to the minimum amount of that nutrient required to support basic function or health within the general population [11]. From this value, governments derive their RDAs/recommended daily intakes (RDIs) [11]. Although this is of clear relevance to (public) health, often in sports nutrition there is less concern with deficiency and more with ‘optimal.’ By this, we are typically referring to nutrient intake at a level to maximize a given parameter related to athletic goals. This involves a more precise attention to diet than is commonplace within the wider population [12], and also more case-by-case interpretation rather than adherence to standardized guidelines (analogous guidelines do not exist for sports nutrition, at least outside the scientific community). Within this review we will consider (potential) nutrient deficiencies, but our emphasis will be on how adopting vegan practices may affect optimal nutrition. We in turn, therefore, define optimal within sports and exercise as dietary approaches that will maximize performance, which we broadly categorize as performance nutrition, adaptation, and recovery.

At face value, placing restrictions upon available foods for the formulation of an athletes’ diet should have a negative impact, purely by narrowing the options for practically delivering nutrients. It is therefore necessary to evaluate key macronutrient and micronutrients present in animal-derived foods that, when removed (or reduced), may impair aspects relating to performance, recovery, or adaptation in the athlete. We then consider whether any highlighted issues can be overcome (or improved) within a well-considered vegan diet. The authors acknowledge that adopting a vegan diet may lead to an increased intake of nutrients such as nitrate and polyphenols [[13], [14], [15], [16]], which are demonstrated to have ergogenic effects when supplemented [17, 18]. However, this review focuses on addressing the potential considerations for the vegan athlete when trying to optimize sports nutrition.

Table 2 collapses data from 20 studies detailing energy and macronutrient intakes in vegans (*n =* 7315) and omnivores (*n =* 165,594) within the general population consuming ad libitum diets, that is, presenting dietary intake differences across vegan and omnivores (within the general population) when left to consume food of their own accord. Due to the lack of such dietary data collected in athletes, it is necessary for some speculation that these differences hold true in athletic populations following a vegan diet without guidance. However, targeted nutritional strategies to optimize macronutrient intake are commonplace within sports nutrition [[19], [20], [21]] and likely means any differences in Table 2 are dampened in athlete populations. Nevertheless, to aid in how best to (if at all) adapt the vegan athlete’s diet, we now use these data as a framework for asking the relevant questions of whether ad libitum and/or more carefully considered vegan diets are likely to help or hinder optimal sports nutrition.

**TABLE 2:** A summary of studies comparing dietary intake between vegan and omnivorous individual.

	*n*		BMI (kg/m^2^)		Energy		Protein		Carbohydrate		Fat	
	OM	VE	OM (kg/m^2^)	VE (kg/m^2^)	OM (kcal/d)	VE (kcal/d)	OM (g/d)	VE (g/d)	OM (g/d)	VE (g/d)	OM (g/d)	VE (g/d)
Haddad et al. [22] (1999)	20	25	25.5	20.5	2064	1957	79.5	63.5	261.0	291.5	78.0	59.5
Allen et al. [23] (2002)	99	94	23.1	22.0	1981	1773	87.2	59.8	243.7	238.5	67.4	60.3
Appleby et al. [24] (2002)	4737	739	24.4	22.3	2046	1813	85.9	59.4	247.1	249.7	71.2	57.8
Larsson and Johansson. [25] (2002)	29	30	21.5	21.8	2714	2524	98.5	63.5	367.0	398.0	87.5	73.0
Davey et al. [26] (2003)	29,913	2112	24.6	22.2	2055	1789	85.8	59.0	244.6	248.2	72.4	55.7
Spencer et al. [27] (2003)	17,824	1553	24.1	22.0	2077	1824	85.7	60.2	246.6	251.7	73.9	57.2
Allen et al. [28] (2008)	25	46	23.3	21.7	2088	1782	91.5	58.8	254.2	234.7	74.5	62.9
Gilsing et al. [29] (2010)	226	232	26.1	22.7	2533	2031	104.5	64.5	276.1	271.1	95.7	67.5
Crowe et al. [30] (2011)	1359	87	25.0	22.3	2007	1768	87.3	56.6	241.3	243.1	70.7	57.8
Schmidt et al. [31] (2013)	168	167	24.9	22.4	2210	1912	85.6	61.2	281.8	274.4	78.1	62.5
Bradbury et al. [32] (2014)	168	167	24.9	22.4	2210	1912	88.4	62.1	265.2	258.1	78.6	61.6
Clarys et al. [33] (2014)	155	104	—	—	2985	2383	112.0	82.0	322.0	336.0	122.0	68.0
Kristensen et al. [34] (2015)	1627	75	24.5	21.0	2225	2432	94.2	75.5	258.7	276.8	88.8	75.9
Schmidt et al. [35] (2015)	95	96	24.4	22.1	2198	1828	82.3	57.8	281.9	254.1	78.1	61.7
Elorinne et al. [36] (2016)	19	22	22.6	21.9	2173	2149	103.0	74.0	182.0	252.0	109.0	88.0
Schmidt et al. [37] (2016)	98	98	24.4	22.1	2183	1828	81.0	57.0	279.0	254.0	77.0	61.7
Sobiecki et al. [38] (2016)	18,244	803	—	—	2089	1942	89.0	63.0	250.5	262.0	72.0	65.8
Allès et al. [39] (2017)	90,664	789	—	—	1899	1877	80.7	62.0	199.6	235.7	78.4	72.7
Pinto et al. [40] (2017)	24	23	23.3	23.5	1952	1833	81.0	60.9	240.1	258.9	73.3	62.9
Schüpbach et al. [41] (2017)	100	53	23.0	21.6	2319	2469	85.0	64.0	259.0	324.0	94.0	96.0
**Mean**			**24.1**	**22.0**	**2101**	**1991**	**89.4**	**63.24**	**260.1**	**269.9**	**82.0**	**66.4**

OM, omnivore; VE, vegan.

### Energy Intake

Energy requirements arguably form the theoretical and practical foundation on which most sports nutrition is predicated [42]. Energy requirements within sports nutrition vary markedly with specific sport, body size/composition, sex, training type, season, stage, etc all playing a significant role [42]. Regardless of absolute energy requirements, failure to reach energy balance, or conditions of prolonged low energy availability [17], can rapidly result in an increased risk of injury, illness, overtraining syndrome, and (undesired) weight loss (including muscle mass) [14,19], impairing training adaptation and performance [13,16,17]. Although failure to maintain energy balance is not an issue for many athletes, it might be of concern in larger athletes and/or those taking part in particularly high volume/intensity training [18,43]. Commonly reported reasons for this are limited time, difficulties consuming large volumes of food, frequent travel, and consequent limited access to food [14]. In contrast to meeting high calorie intakes, consideration is also required for those limiting energy intake as is frequently the case for athletes competing in weight restriction sports (eg, boxing, body building, horse racing) or during periods of reduced activity (eg, out of season, injury).

Animal-derived foods comprise a large proportion of energy intake in the omnivorous diet. For context, in the example diet we provide for a strength athlete (Supplementary Table 1), 43% of total energy intake comes from animal sources. Furthermore, a vegan diet is high(er) in fruits and vegetables [33,44], which typically contain less energy for a given mass of food (ie, lower energy density foods). The typically higher fiber content of plant-based foods also promote satiety [45]. Although these foods can be useful in facilitating weight loss [46] and can be useful for athletes looking to reduce calorie intake, low energy density foods that promote early satiety could make it difficult for athletes to meet high energy targets. Furthermore, foods high in fiber may promote gastrointestinal stress during and after exercise [47]. No comprehensive assessments are available examining athletes’ ad libitum energy intakes when animal-based products are removed from the diet. However, a recent systematic review concluded that in the general population, individuals following a vegan diet consume fewer calories compared with individuals following omnivorous/vegetarian diets [48]. In line, Table 2 shows that all [33,[22], [23], [24], [25], [26], [27], [28], [29], [30], [31], [32], [34], [35], [36], [37], [38], [39], [40], [41]] bar 2 [34,41] of the studies reported that vegans consumed less energy compared with omnivores, with a peak daily difference of 2.5 MJ (602 kcal) between populations [33]. However, when averaged across studies, the energy intake of vegans was only 5% lower compared with their omnivorous counterparts, a difference that is likely inconsequential considering that vegan individuals often have a lower body mass [5]. Therefore, it appears that athletes looking to adopt a vegan diet can do so without compromising energy balance, although they should bear it in mind as a consideration, especially under conditions of particularly high energy demand.

## Macronutrients

### Protein

Although the provision of energy to support athletic training and performance does not appear to represent a major concern for the vegan athlete, Table 2 does demonstrate a clear shift in the proportion of energy being provided by each macronutrient. The most obvious and well documented difference in macronutrient intake between vegans and omnivores concerns dietary protein. Skeletal muscle mass is regulated through dynamic fluctuations in muscle protein synthesis (MPS) and muscle protein breakdown rates. Prolonged periods where MPS exceeds muscle protein breakdown will result in the net muscle protein accretion, and periods where muscle protein breakdown exceeds MPS will result in the loss of muscle mass, with changes in MPS thought to be most consequential [49]. Irrespective of net balance, the dynamic process of muscle protein turnover broadly governs the multifaceted nature of adaptive responses (to training) and, thus, the changing muscle phenotype. The provision of dietary protein stimulates MPS, as the transient increase in circulating amino acids following protein ingestion act as both stimulus and substrate for the accretion of muscle protein [50]. Muscle contraction in the form of resistance- or endurance-type exercise provides additional stimulus to upregulate MPS in both an additive and independent manner [[51], [52], [53]]. As such, it is the combination of prolonged strategic protein feeding and structured and progressive exercise training that permits chronic muscle remodeling. Given the central role of dietary protein in this process, consideration should be given to whether the vegan diet provides optimal protein intake, which we will consider from the perspectives of quantity and quality consumed.

#### Quantity

The current UK/US RDAs/RDIs for dietary protein intake in the general population are 0.75 to 0.8 g/kg body mass [BM]/d [20,54]; however, there is scientific consensus that athletes of all persuasions require higher intakes to optimize training adaptations, recovery, and/or performance [16,55,56]. Depending on training volume, dietary recommendations for endurance athletes range from ∼1.2 to 1.6 g/kg BM/d to support recovery, promote training adaptations, and account for the contribution of elevated amino acid oxidation rates during exercise [55,[57], [58], [59]]. Similarly, recent meta-analyses indicate resistance training athletes of all levels require ≥1.6 g/kg BM/d to optimize training-induced increases in muscle mass and strength [60]. Furthermore, for the majority of athletes’ training modalities are not mutually exclusive, as most athletic training programs will incorporate both endurance and resistance training, perhaps increasing protein requirements further. In support, a recent systematic review suggests protein intakes of 1.7 to 2.2 g/kg BM/d to support lean mass adaptation during concurrent exercise training [61]. A caveat to these guidelines is that they have been generated based on data accumulated in omnivorous individuals. Whether or not vegan athletes consume sufficient dietary protein and, if not, whether they *can*, has rarely been considered. This is consequential given the potential for this to result in altered dietary protein guidelines for vegan athletes.

Animal-based food sources are generally protein dense, especially meat and dairy products, and therefore comprise a major proportion of protein intake in the general population [23,37,39]. In contrast, plant-based protein sources typically contain a much lower proportion of protein. This typically necessitates larger quantities of plant-based food to be consumed to obtain a comparable serving of protein. The summary of studies we present in Table 2 reliably demonstrates (in all studies included) that vegans within the general population consume considerably (∼30%) less protein compared with omnivores. This suggests that ad libitum dietary protein intake is markedly lower (and largely suboptimal from a sports nutrition perspective) in most individuals who adopt a vegan diet. However, multiple studies [[62], [63], [64]] have shown that planning and adhering to a high(er) protein vegan/vegetarian diet is eminently achievable, which we illustrate in sample diets where consideration to food choices can achieve protein intakes in line with current recommendations (Supplementary Table 1 and Supplementary Table 2). Emerging data and commercialization around novel alternative protein-rich foods will likely aid in this process from a practical perspective.

#### Quality

The term ‘protein quality’ is difficult to succinctly define and is discussed in more detail elsewhere [7,65]. Briefly, the term encompasses a protein source’s amino acid composition, digestibility, and subsequent bioavailability of (specific) amino acids, and the metabolic fate of those amino acids. Such factors are affected by the specific protein source, whether consumed as isolated protein or as a protein-rich whole food, and whether that protein source is consumed alongside other foods (ie, a meal). Irrespective, the quality of a protein source has reliably been shown to play an important role in determining the magnitude of the postexercise MPS response. For example, milk, egg, and meat-derived proteins all stimulate robust postexercise MPS responses [[66], [67], [68]]. This has been attributed to their high essential amino acid (EAA) content [69], particularly leucine [70], a lack of any notable amino acid deficiencies [71], rapid digestibility [72], and a high total digestibility/absorbability and therefore availability in the circulation (ie, ‘bioavailability’) [72]. To illustrate these concepts, Gorissen et al. [72] collated multiple studies using intrinsically labeled milk proteins and demonstrated that ∼65%, 57%, and 45% of the protein from milk, whey, and casein becomes available in the circulation, respectively, over a ≥ 5-h postprandial period. Similarly, van Vliet et al. [68] showed, over a 5-h postprandial window, that ∼67% of the protein becomes available following the consumption of intrinsically labeled egg protein. Furthermore, Pennings et al. [73] observed ∼61% and ∼49% availability of amino acids from minced beef and beef steak over a 6-h postprandial period, respectively. It is from data using such animal-based protein sources that we draw much of our understanding on optimal protein nutrition such as dose [74,75], timing, [76,77]. and daily distribution [76].

In contrast, fewer in vivo data covering (postexercise) MPS responses following the ingestion of non–animal-derived proteins are available. Nevertheless, there is the widely held view that nonanimal proteins are inferior with respect to their capacity to stimulate postexercise MPS rates compared with animal proteins. The lower anabolic potential of non–animal-derived proteins is thought to be attributed to a few factors. The presence of nonprotein constituents and antinutritional factors are thought to slow (and reduce) the digestion and absorption of protein, meaning that a lower proportion of ingested amino acids become available (at a reduced rate) in the circulation postprandially [78,79]. However, differences in protein absorbability are ameliorated after protein purification through removal of nonprotein constituents to produce protein isolates [[80], [81], [82]]. This suggests that the lower digestibility of non–animal-derived protein is attributable to their food matrix, rather than the protein per se*.* Although the protein found in non–animal-derived sources may be equally digestible, plant sources frequently have lower total EAA contents, and leucine, methionine, and/or lysine in particular, which have been suggested to provide limitations for MPS at either the molecular signaling or substrate availability levels [83].

Studies comparing the MPS response following wheat or soy protein ingestion to animal-based sources support the above narrative [[84], [85], [86]]. For example, Wilkinson et al. [84] demonstrated that 18 g of soy protein stimulates MPS to a lesser extent compared with an isonitrogenous, isoenergetic, macronutrient-matched bolus of milk protein. Further, casein ingestion resulted in a greater increase in MPS compared with a protein-matched wheat protein hydrolysate in healthy older men, as wheat failed to significantly increase rates of MPS above postabsorptive levels [87]. However, there is a growing number of studies that contradict, or at least obfuscate, this narrative [53,[88], [89], [90], [91]]. For example, postprandial rates of myofibrillar and mitochondrial protein synthesis were equivalent following the ingestion of soy and whey protein (coingested with carbohydrate), following a bout of concurrent training in young men [53], and no differences in postprandial MPS rates were observed between isonitrogenous (30 g) servings of milk and wheat protein in young men [89]. We have recently demonstrated that bolus ingestion of mycoprotein, a fungal-derived protein-rich food source, resulted in greater stimulation of MPS compared with a leucine-matched bolus of milk protein [91]. These data suggest that nonanimal protein sources are not necessarily less anabolic compared with animal-based sources but require consideration on a case-by-case basis. An emerging theme is that differences between animal- and plant- based sources are absent when higher doses of protein (presumably obviating any individual amino acid deficiencies) are compared (∼30 g protein), though this is not always the case [53,86]. Therefore, although 20 g may be considered optimal for animal-based proteins (with larger doses resulting in a sharp increase in amino acid oxidation) [74,75], a higher dose may be needed for (some) nonanimal proteins. Clearly, more research is needed to identify sources and source-specific doses to optimize (or maximize) the response in exercised muscle. Perhaps a pressing pragmatic concern, however, is how to optimize efficiency of potentially suboptimal protein sources, given that simply consuming more will not always be advisable or possible.

Blending plant-based protein sources is a suggested method to obviate EAA deficiencies [71,83]. For example, brown rice (eg, a grain) is low in lysine but high in methionine, whereas pea (eg, a pulse) is low in methionine and high in lysine [7,77]. As such, the combination of these 2 sources would result in a more complete EAA profile. A handful of studies have supported the blending theory by demonstrating that a plant/animal protein blend [89,90,92,93] or exclusively plant protein blend [88,94,95] can stimulate an equivalent MPS response to an animal-based comparison. However, a caveat to the plant protein blend studies is that all were fed ≥30 g of protein, therefore, any EAA deficiencies corrected by the blending are also possibly already accounted for by the relatively large bolus of protein.

Another suggested method to improve the anabolic potential of plant proteins is to fortify a given protein with amino acids. Leucine is considered the key amino acid for activating the molecular signaling cascade that underpins the postprandial increase in MPS [96]. Plant proteins are often low in leucine [80], and it is thought that additional leucine might potentiate their postprandial anabolic response. In line, leucine fortification has been shown [70,97,98], though not always [99], to augment the MPS response to suboptimal doses of animal protein. However, evidence to date shows no benefit of fortifying non–animal-derived protein sources with leucine [100] or branch chain amino acids [101], suggesting that other amino acids may be limiting to the MPS response. Lysine fortification has been demonstrated as an effective method of supporting growth and lean mass accrual [[102], [103], [104]]. A single study has investigated a lysine-enriched plant-based protein blend and demonstrated parity with a chicken comparison with respect to its ability to increase MPS in humans [94]. This study also used a relatively high dose of protein (≥30 g) and did not include a non–lysine-enriched control. Clearly, more research is needed to establish whether amino acid fortification is an effective or necessary method of improving the anabolic potential of plant proteins.

The major question is to what extent the lower quality of certain non–animal-derived proteins requires a greater protein intake and/or whether selection of higher-quality sources, blending, and/or fortification can mitigate this within a vegan diet. When translating to applied nutrition, further research is required; for example, establishing dose–MPS response relationships of different proteins, the role of the whole food matrix (plant and animal), ecologically valid, and varied exercise paradigms and importantly, how such factors ultimately translate to animal and nonanimal diets supporting phenotypic change over days, weeks, and months.

#### Chronic studies

Longer-term diet and exercise intervention studies relevant to vegan protein sources and changes in body composition are mainly restricted to supplemental protein-only studies, thereby addressing the utility of a single protein source. Such studies have produced equivocal results, with some [105,106] observing greater training-induced increases in lean mass when supplementing with animal proteins (whey or milk) compared with nonanimal alternatives (soy), whereas others [[107], [108], [109], [110]] have found no differences when whey has been compared with rice, pea, soy, or a pea/yeast protein blend. The disparity may be explained by the amount of protein that was supplemented, whereby supplementation of sufficient protein obscures any differences when less is consumed. Broader dietary control studies show a similar trend. For instance, greater training-induced lean mass gains were reported in participants consuming an omnivorous diet compared with those consuming a lacto-ovo-vegetarian diet at 0.78 g/kg BM/d [111], but these differences were no longer evident when protein intakes increased to 1.15 g/kg BM/d [112]. In line, recent work showed identical hypertrophic adaptations during twice weekly lower limb resistance training between habitual omnivores and vegans consuming 1.6 g/kg BM/d [62], findings that held true in our own work where a vegan dietary intervention was comparable with an omnivore’s diet in support of hypertrophy and strength during whole-body resistance training [64].

Available evidence to date indicates that vegan athletes consuming sufficient dietary protein are unlikely to experience any decrement to adaptive responses during training. In this instance, *enough* and *optimal* are essentially interchangeable; although it is feasible that optimal daily protein intake could be (modestly) higher for the vegan athlete, to what extent is not clear and is likely modulated by the quality of the vegan protein sources selected. As with protein amount, selection of protein sources for the vegan athlete likely offers the pragmatic challenge (with supplemental isolates, concentrates, or hydrolysates more attractive) of requiring more consideration and planning than for the omnivorous athlete.

### Carbohydrate

Carbohydrates are the primary fuel source for moderate to vigorous exercise as they are rapidly metabolized via anaerobic (glycolysis) or aerobic (TCA cycle/oxidative phosphorylation) pathways to (re)synthesize ATP at sufficient rates [113,114]. The endogenous production and carbohydrate reserve, stored as plasma glucose or glycogen within muscle and liver, is relatively small, and depletion is detrimental to performance [15,115]. Therefore, sufficient daily intakes and provision of carbohydrate before and sometimes during exercise are essential to ensure optimal performance [15,116]. Furthermore, tournament or multiday/stage sports (eg, Tour De France) necessitate carbohydrate ingestion postexercise to optimize recovery and ensure optimal endogenous carbohydrate availability for subsequent efforts [15]. In addition to its role as a substrate, glycogen stores modulate various cell signaling pathways underpinning training adaptations [117]. There is evidence to suggest that performing endurance training with depleted muscle glycogen stores upregulate such molecular pathways linked with greater endurance adaptation (e.g., mitochondrial biogenesis) [118]. Alternatively, performing resistance exercise with low muscle glycogen, and therefore low cellular energy status, can blunt the signaling pathways responsible for hypertrophic adaptation [119,120]. Clearly, the decision on whether to train in a glycogen depleted or loaded state will be determined by the specific goals/training phase of the athlete [121].

As demonstrated in Table 2, the lower energy intake from dietary protein within the vegan population is exclusively compensated for by increased carbohydrate intake, a finding true of all-bar-one of the studies we examined. As such, risk of deficient or suboptimal total carbohydrate intakes in the vegan athlete seems unlikely. However, worthy of consideration is that specific types of carbohydrate not present in plant sources may be lacking in the vegan athlete’s diet, specifically, lactose and galactose given their high prevalence in dairy products [122]. Although lactose and galactose are metabolized differently to glucose and fructose [123, 124], consumption of lactose and galactose confers no additional benefits to time trial performance [125,126], and in the case of galactose, may even be detrimental (compared with other monosaccharides) [125]. However, a possible glycogen sparing benefit of lactose compared with sucrose ingestion during exercise has been observed [126]. The role of ingesting galactose and lactose alone (or in combination) in facilitating the rate of muscle glycogen recovery following exercise is yet to be investigated. However, coingesting galactose with maltodextrin resulted in comparable postexercise liver glycogen repletion to a fructose/maltodextrin condition, and double that of a glucose-only condition [127], likely due to the necessary hepatic metabolism of galactose. Data so far therefore demonstrate minimal benefits of milk-derived carbohydrate over and above what is available from plant-based sources for athletic performance, recovery, and/or adaptation. Therefore, the quantity and type of carbohydrate available in the vegan diet appears sufficient and optimal to meet the needs of the athlete.

Though athletes can likely fulfill the metabolic requirements for total and type of carbohydrate intakes while following a vegan diet, it is worth highlighting some practical considerations. Vegan diets tend to be higher in fiber, in large part due to the type of carbohydrates consumed in more bulk, and fiber has been linked to the promotion of satiety [45]. Although this may not impact overall energy intake, as established above, it could be inhibitory when needing to refuel in a short time period (multistage events, etc). Therefore, simple choices should be made when ingesting high quantities of carbohydrate in these short feeding windows, such as the selection of foods low in fiber (rice, pasta, noodles, etc), as opposed to higher fiber alternatives (oats, whole grains, beans, etc). A final consideration would be the potential for the plentiful carbohydrate intake of a vegan athlete to make ‘train low’ (ie, low carbohydrate availability training), in an effort to drive adaptation, more challenging. As a result, the athlete/practitioner will need to carefully consider training needs and personal preferences if wishing to exploit this strategy under vegan conditions.

### Fat

Dietary fat is often perceived negatively due to the adverse health effects associated with its overconsumption. However, lipids play many key roles within the body. Specifically for athletes, fat is a rich energy source (∼9 kcal/g) compared with carbohydrates and protein (∼4 kcal/g). However, the breakdown of fat for energy via β-oxidation is exclusively reliant on the availability of surplus cellular oxygen, making it a less efficient source of ATP generation during exercise compared with carbohydrate. As a result, the relative contribution of fat oxidation to meet energy demand decreases as exercise intensity increases [114]. As such, although fat stores can be a central energy source for low intensity, long duration endurance exercise, exercise performance beyond these intensities is heavily reliant on the finite carbohydrate stores. Beyond its role as an energy substrate, dietary fat also plays more specific roles [14,128] that will be discussed below.

#### Total fat intake

The vast majority of studies have concluded that vegan individuals consume less total fat compared with omnivores (Table 2). Indeed, much of the purported health benefits associated with adopting vegan diets have been attributed to lower (saturated) fat intakes [26]. Unlike carbohydrate stores, fat stores are considered an essentially infinite energy store that are unlikely to bring about limitations to performance even during the most prolonged exercise bouts. Therefore, the typically lower intakes of fat in vegans compared with omnivores is unlikely to present a concern from an energy availability perspective. However, whereas adipose tissue may be plentiful, site-specific stores such as intramuscular triglycerides (IMTGs) are not. IMTGs are a key source of energy during exercise (especially for the trained endurance athlete) as they are more readily oxidized for the regeneration of ATP compared with peripheral stores [129]. Nevertheless, Coyle et al. [130] demonstrated that a diet with just 22% of energy coming from fat did not reduce IMTG stores. In fact, it was not until dietary fat intake was limited to 2% of total energy intake that significant reductions in IMTG stores occurred [130]. Although the fat content of a vegan diet does not appear to compromise exercise performance, diets low in dietary fat could theoretically impair absorption of fat-soluble vitamins or reduce sex hormone concentrations [131]. However, there is currently no evidence suggesting that vegans are deficient in the fat-soluble vitamins, and comparisons of circulating testosterone between vegans and omnivores have revealed no differences [132].

#### Type of fat intake

If total dietary fat intake is of little concern to the vegan athlete, it is worth pondering whether intakes of different *types* of fat may be of relevance. Dietary fat exists within a variety of (sub)categories (ie, carbon chain length, saturation status, etc) which are metabolized differently and, as a result, can fulfill diverse and distinct roles. Vegans consume lower amounts of cholesterol and conjugated linoleic acid (CLA) [33,26]. Despite cholesterol performing many key roles within the body [133] and some early in vitro evidence to suggest that cholesterol can act on anabolic signaling cascades [132], there are no data to suggest that vegans are deficient (or suboptimal) to the point where it will impact any aspect of performance, adaptation, or recovery. Likewise, there are no data to suggest that vegans are deficient for CLA, and further, whether this is likely to affect the athlete. Moreover, despite compelling evidence that CLA supplementation can influence body composition in rodent models [134,135], human data are more modest [[136], [137], [138]].

Vegans consume greater amounts of PUFAs compared with omnivores [48]. PUFAs can be further subdivided, with plant-based diets tending to be rich in n-6 PUFAs, which are found in high amounts in nuts, seeds, soybeans, tofu, and corn. Conversely, foods rich in n-3 PUFAs are limited in the vegan diet (with the exception of α-linolenic acid, which is rich in plant oils) and are mainly present in appreciable amounts in marine-based sources, with 2 specific n-3 PUFAs (EPA and DHA) almost exclusively available in the Western diet from fish oils. The lower n-3 PUFA intake in vegans is reflected in reduced circulating concentrations compared with omnivores [33,26,139]. This tight link is likely explained by n-3 PUFA endogenous synthesis being inefficient, limited by the low activity of the rate-limiting enzyme in n-3 PUFA biosynthesis, omega-3 desaturase. As a consequence, n-3 PUFAs are considered a conditionally essential nutrient. Of interest, most individuals, irrespective of dietary habits, fail to meet the recommendations for n-3 intake (200–500 mg/d) [140]; however, this is likely to be exacerbated in the vegan athlete. Studies are investigating whether n-3 supplementation can improve parameters of adaptation, performance and recovery. With respect to strength based activity, studies to date have shown that n-3 supplementation can potentiate the MPS response to hyperaminoacidemia–hyperinsulinemia [141,142] and augment muscle adaptive responses during prolonged training in older adults [143,144], with suggestions that the causative mechanism(s) may be anti-inflammatory. However, such data have not translated yet to increased MPS responses or augmented hypertrophy in younger, more trained, and/or exercising populations, especially when sufficient protein is also provided [145]. Regarding endurance based sports, n-3 supplementation has been reported to positively affect parameters of oxidative capacity [146,147] and oxygen efficiency [148,149] but this also has not translated to any meaningful improvements in endurance exercise performance [150]. Overall, the current data suggests that n-3 supplementation does not translate to any benefit to the athlete, though an exception may be during injury. McGlory et al. [151] found that n-3 supplementation significantly attenuated the loss of skeletal muscle size and leg lean mass during single limb immobilization. This attenuation of muscle loss during the immobilization expedited a full recovery of skeletal muscle volume after 2 wk of returning to habitual physical activity.

Taken together, current evidence suggests that vegans consume lower amounts of dietary fat, but higher amounts are not necessary, as normal vegan intakes are unlikely to limit energy availability during exercise or, therefore, performance, adaptation, or recovery. Although specific fats, such as n-3 PUFAs, are consumed in lower quantities with associated impacts on related biomarkers in vegans, concrete human evidence as to any detriment of this on performance, recovery or adaptation is currently lacking. Supplementation, therefore, although an option, does not seem to be required.

## Ergogenic Aids

### Creatine

Creatine is a nonproteogenic compound synthesized in the liver and pancreas from the amino acids arginine and glycine [152]. The main store of creatine in the body is found in skeletal muscle (>95%) [153], where roughly two-thirds is stored as phosphocreatine (PCr) and one-third exists as free creatine [154]. Creatine plays a central role in maintaining ATP availability during high intensity, anaerobic exercise and/or in the transition from rest to exercise (or increasing exercise intensities) when a degree of metabolic inertia is present. As ATP is broken down to ADP to provide rapid energy delivery for high-intensity muscle contraction, the free energy released from the hydrolysis of PCr to creatine + inorganic phosphate (catalyzed via the creatine kinase enzyme) is used to rapidly resynthesize ATP. The creatine kinase/PCr system also acts as an energy shuttling system, connecting sites of energy production (glycolysis and oxidative phosphorylation in the mitochondria) to subcellular sites of ATP utilization to fuel energy metabolism [155].

Harris et al. [156] demonstrated that 4 to 6 doses of 5 g of creatine per day for 5 to 7 d increased muscle creatine content by an average of 17% (a range of 0 to 40%). Following early anecdotal reports that creatine supplementation may improve exercise performance [157], further work translated nutritionally induced muscle creatine loading to show an exercise performance-enhancing effect [158]. Since, many studies have corroborated the benefits of creatine supplementation on repeated bouts of high-intensity exercise [[159], [160], [161], [162], [163], [164]], attributed to reduced ATP degradation during exercise and improved ATP resynthesis during the recovery periods [165]. Creatine supplementation has also been demonstrated to potentiate muscle hypertrophy, strength, and muscle fiber cross-sectional area growth in response to resistance training [166,167]. It is believed that the potentiated response to resistance training is facilitated by increased intensity of individual workouts as a result of better matching between ATP supply and demand during exercise [[168], [169], [170]]. There is also evidence that creatine supplementation can improve recovery from exercise, via enhanced muscle glycogen replenishment [171,172], attenuated muscle damage [173], inflammation, and muscle soreness [174], and that it can improve cognitive function [175,176]. This wealth of evidence promoting the efficacy of creatine supplementation has led the International Society of Sports Nutrition to conclude that, for athletes aiming to increase high-intensity exercise capacity and lean body mass, creatine is the most effective ergogenic nutritional supplement currently available [154].

Creatine supplementation might be particularly auspicious for vegan athletes. Roughly 1% to 2% of the body’s creatine pool (∼2 g for the average 70 kg male) is excreted per day as creatinine [177]. Therefore, for the average 70 kg male, the daily requirement for creatine is ∼2 g, 50% of which is obtained through the diet [178]. However, creatine is only available in animal products, primarily meat (i.e., muscle tissue) and, albeit less so, from dairy products. Due to the lack of creatine in the vegan diet, vegan and vegetarian individuals have lower plasma (30% to 50%) [179,180] and intramuscular (∼12%) [181] creatine levels compared with omnivores. It is currently unclear whether the lower creatine concentrations observed in vegans results in impaired performance or hypertrophy. However, given that creatine supplementation can have profound effects on parameters of adaptation and performance, it seems logical that vegans may experience a decrement in this regard, and may wish to consider supplementing with creatine, particularly as it has previously been demonstrated that creatine supplementation resulted in greater increases in creatine stores and lean mass in vegetarians compared with omnivores [181].

Creatine is clearly a supplement worth considering for vegan athletes who compete and train at high exercise intensities and/or are looking to maximize hypertrophic/strength adaptation. Athletes competing in events <30 s (100–200 m sprints), sports containing repeated sprints (eg, football/netball), or sports where high muscle mass are crucial will benefit most from taking creatine. The most common creatine dosing strategy involves a loading phase, consuming 5 g (or 0.3 g/kg body weight) 4 times daily for 5 to 7 d in order to saturate muscle creatine stores. This can then be maintained by ingesting 3 to 5 g/d, although larger athletes may need to ingest more (5 to 10 g/d) [154,156,182]. Alternatively, to avoid the initial loading phase and the associated water retention, 3 g/d can achieve the same level of creatine store increase. Finally, athletes should look to consume their creatine with carbohydrate and/or protein to optimize muscle creatine uptake (in the form of a carbohydrate/protein-containing drink or in close proximity to a meal).

### Carnitine

Carnitine is a naturally occurring, conditionally essential nutrient that is primarily found in mammalian tissue. Roughly 75% of carnitine in the body is obtained through the diet, mostly in meat [183]. More than 90% of the body’s carnitine store resides in skeletal muscle where it performs 2 key roles in energy metabolism [184]. First, carnitine acts as a transporter of long chain fatty acids across the mitochondrial membrane for β-oxidation. The formation of acylcarnitine (acyl-CoA + carnitine) via carnitine palmitoyltransferase allows acyl-CoA to cross the otherwise impermeable inner membrane and enter the mitochondrial matrix for oxidation. The second role of carnitine is as a buffer for excess acetyl-CoA production from glycolysis by forming acetylcarnitine, particularly necessary during high-intensity exercise. This secondary function of carnitine decreases negative feedback from the accumulation of acetyl-CoA and allows continued ATP production from glycolysis and, ultimately, flux through the pyruvate dehydrogenase complex and the TCA cycle to continue.

To date, research investigating the impact of carnitine supplementation on muscle fuel metabolism and/or exercise performance has produced inconclusive results [[185], [186], [187], [188], [189], [190], [191]]. However, it is clear that the lack of performance improvements seen in the majority of these studies can be attributed to a failure to increase muscle carnitine content [[189], [190], [191]]. Carnitine is transported from plasma to the muscle against a high concentration gradient, making increasing muscle carnitine stores difficult in the basal state [192]. However, hyperinsulinemia (>50 mU/L) promotes carnitine uptake into skeletal muscle [192,193] and, therefore, coingestion of carbohydrate with carnitine (94 g and 3 g, respectively) improves whole-body carnitine retention [194] and muscle uptake [50]. In line, chronic (12–24 wk) daily coingestion of carnitine (2 g) and carbohydrate (80 g) twice daily increass the muscle carnitine pool by 21%. Importantly, this increased muscle carnitine availability results in reduced glycogen utilization during low intensity exercise (50% $\dot{V˙}$O_2_ max) (likely due to increased fat oxidation [195]), a reduced contribution of anaerobic ATP production during high-intensity exercise (80% $\dot{V˙}$O_2_ max) (attributed to increased acetyl group buffering), and resultantly increased work output during a 30-min maximal cycling performance test. Supportive data can also be found in similar long-term carnitine coingestion supplementation studies (25 wk of 3 g of carnitine with an insulinogenic beverage containing 44 g carbohydrate and 14 g protein) where ∼20% increases in muscle carnitine content and beneficial shifts in muscle fuel selection were reported in older adults [196]. Collectively, these data suggest that regimented carnitine supplementation, coingested with sufficient carbohydrate and persisted over time, is a viable nutritional approach to bring about beneficial alterations in exercise metabolism and improved exercise performance.

Carnitine homeostasis is primarily achieved via dietary means, with dietary carnitine being obtained from animal-derived sources with only small quantities found in plant-based sources. Omnivores obtain ∼2 to 13 μmol L-carnitine/kg·BM/d exogenously from dietary meat sources, with only ≤1 μmol L-carnitine/kg·BM/d produced by endogenous synthesis from trimethyllysine, and ∼7 μmol L-carnitine/kg·BM/d is excreted, resulting in a relatively tight regulation of total body carnitine content at ∼2 μmol L-carnitine/kg·BM/d [197]. Vegetarians (vegans) typically only consume ∼0.04 to 0.4 μmol∙kg/d of carnitine [197], which results in 20% to 30% lower plasma concentrations compared with omnivores [197,198], although this does not fall outside the normal limits [199]. Some [198], but not all [200], studies also report that vegetarian individuals have a lower muscle carnitine content compared with omnivores. However, these studies did not report any alteration in muscle fuel utilization or exercise performance, and so a quantifiable level of muscle carnitine ‘deficiency’ in the context of optimal sports nutrition is not yet identifiable. However, some critical threshold is likely given the effects of severe carnitine deficiency can be clearly seen in patients with carnitine transporter deficiency (muscle carnitine content of less than 1% of normal [201,202]), who experience excessive muscle pain and weakness, hypoglycemia, and myoglobinuria during prolonged exercise [203, 204].

A single study has investigated the effects of carnitine supplementation on performance outcomes in vegetarian individuals. Novakova et al. [200] investigated whether 12-wk of carnitine supplementation would impact muscle carnitine stores and muscle metabolism/function in healthy male omnivores and vegetarians. At baseline, the vegetarian group were found to have lower plasma carnitine concentrations compared with omnivores. However, in contrast with previous findings [199], muscle carnitine levels were not different between populations, and neither were performance outcomes. The 12-wk supplementation protocol (1 g twice daily with morning and evening meals) significantly increased muscle carnitine stores in the vegetarian group (11%), but not the omnivore group, underscoring the potential for supplementation in the vegan athlete. However, contradictory to the findings of Wall et al. [205], this increase in muscle carnitine content had no impact on muscle fuel metabolism (respiratory exchange ratio (RER) at exhaustion or blood lactate/RER during 1 hr of exercise at 75% $\dot{V˙}$O_2_ max) or exercise performance ($\dot{V˙}$O_2_ max, maximal physical capacity). This is perhaps explained by the fact that Novakova et al. saw a smaller increase in carnitine content (13% compared with 21%), which is likely attributable to the shorter supplementation period and/or lower dose.

Irrespective of the lack of clarity over deficiency in vegans, the ability of carnitine supplementation to augment performance makes it a worthwhile consideration for the vegan athlete. One practical consideration merits discussion. For carnitine supplementation to successfully increase muscle carnitine stores, it must be coingested with sufficient carbohydrate to ensure an increase in circulating insulin concentrations >50 mU/L. This may have implications for energy balance. However, if the athlete is already consuming high carbohydrate snacks/meals, supplementing carnitine with these high carbohydrate feedings is relatively simple. Future research may focus on establishing lower calorie/alternative mechanisms to facilitate muscle carnitine uptake. Recommendations from the European Food Safety Authority suggest that up to 2 g/d of carnitine is safe [206]. However, studies to have demonstrated a significant increase in muscle carnitine stores in omnivores have used doses >3 g/d [195,196,205]. However, 2 g/d carnitine supplementation for 12 wk successfully increased muscle carnitine stores in vegetarian individuals [200]. To date, no adverse events have been reported with carnitine supplementation for up to 6 mo [196], however, less is known beyond that time frame. Therefore, targeted/periodized supplementation to increase muscle carnitine for specific events (or time periods) is preferred to constant supplementation. More will be known about this when the maintenance/washout of carnitine loading protocols has been better defined.

### Carnosine

Carnosine (β-alanyl-L-histidine) is a dipeptide found in high concentrations (20–30 mmol/kg in dry weight) within skeletal muscle where it primarily acts as a buffering agent. Initial studies identified a carnosine side-chain pKa of 6.83, making it an effective buffer for times where the typical physiological cellular pH range is exceeded (ie, very high-intensity exercise). Skeletal muscle carnosine synthesis, catalyzed by carnosine synthase, is contingent upon the presence of its 2 precursors, histidine and β-alanine. Histidine is abundant within skeletal muscle; however, β-alanine is found in relatively low concentrations, driven by the high *K*_m_ for carnosine synthase and the subsequent rapid formation of carnosine [207,208]. Therefore, working on the assumption that beta-alanine is rate limiting to carnosine synthesis *in vivo*, studies have investigated whether β-alanine supplementation is capable of increasing muscle carnosine content and, as a result, improving (high intensity) exercise performance.

β-alanine supplementation has been demonstrated to increase muscle carnosine stores in untrained [209] and trained individuals [210]. Hill et al. [211] extended on these findings by showing 10-wk of β-alanine supplementation increased muscle carnosine content and resulted in an improvement in a cycling capacity test at 110% W_max_. Many studies have since investigated whether β-alanine supplementation can improve exercise performance and have observed mixed results [[212], [213], [214], [215]], with the greatest effects in exercise lasting 1 to 4 min [216]. Furthermore, there is some evidence that β-alanine supplementation can improve intermittent exercise performance [216] and therefore may be auspicious for games athletes. There are also data to suggest that β-alanine supplementation alongside training may, but not always [217], augment training adaptations over simply training alone [218]. Collectively, therefore, β-alanine supplementation is worth considering for athletes competing in high intensity, moderate duration (1–4 min) exercise. However, there is a need to determine more sport-specific improvements.

β-alanine is exclusively found in meat and fish and is therefore completely absent from the vegan/vegetarian diet. Given the tight link between β-alanine intake and muscle carnosine content, it is no surprise that vegetarians have lower muscle carnosine contents compared with omnivorous individuals [219,220]. Whether these lower muscle carnosine concentrations result in impairments in exercise performance is yet to be established. However, given that increasing muscle carnosine content can improve exercise performance [216], it is plausible that reduced muscle carnosine content can impair performance. Therefore, akin to the advice on creatine and carnitine, most athletes stand to improve adaptation and/or performance from β-alanine supplementation. Athletes who follow a vegan diet may be particularly inclined to supplement with β-alanine to also mitigate any impairments that may be associated with the lower muscle carnosine concentrations. Dosing strategies that have successfully increased muscle carnosine content in trained individuals is as follows: daily doses consisting of 2.4 g/d during the first 4 d, 3.6 g/d during the subsequent 4 d, and 4.8 g/d thereon. It is advised to split this into smaller doses (400 mg) that are regularly ingested throughout the day (∼2 h between each dose) to prevent symptoms of paresthesia (a prickling sensation on the skin).

### Micronutrients

In addition to optimizing intakes of energy, macronutrients, and specific metabolic compounds relating to fuel metabolism, an often underappreciated consideration within sports nutrition is micronutrient (vitamins and minerals) status. Large cohort studies indicate that vegans have a higher risk (compared with omnivores) for deficiencies in several micronutrients predominantly found in animal-derived foods, such as vitamin B12, iron, calcium, and vitamin D [1,26,[1], [37], [38], [39], [40], [41], [49], [50], [51], [52], [53], [54], [55], [56], [57], [58], [59], [60], [61], [62], [63], [64], [65], [66], [67], [68], [69], [70], [71], [72], [73], [74], [75], [76], [77], [78], [79], [80], [81], [82], [83], [84], [85], [86], [87], [88], [89], [90], [91], [92], [93], [94], [95], [96], [97], [98], [99], [100], [101], [102], [103], [104], [105], [106], [107], [108], [109], [110], [111], [112], [113], [114], [115], [116], [117], [118], [119], [120], [121], [122], [123], [124], [125], [126], [127], [128], [129], [130], [131], [132], [133], [134], [135], [136], [137], [138], [139], [140], [141], [142], [143], [144], [145], [146], [147], [148], [149], [150], [151], [152], [153], [154], [155], [156], [157], [158], [159], [160], [161], [162], [163], [164], [165], [166], [167], [168], [169], [170], [171], [172], [173], [174], [175], [176], [177], [178], [179], [180], [181], [182], [183], [184], [185], [186], [187], [188], [189], [190], [191], [192], [193], [194], [195], [196], [197], [198], [199], [200], [201], [202], [203], [204], [205], [206], [207], [208], [209], [210], [211], [212], [213], [214], [215], [216], [217], [218], [219], [220], [221], [222], [223], [224], [225], [226]]. Although the impact of micronutrient status on key measures of exercise performance or adaptive responses to training is minimal (in comparison with macronutrients), clearly any deficiencies play a role in overall health. although athlete health is largely beyond the scope of the present review, any deleterious health effects are likely to disrupt training volume, impacting adaptation and performance. Therefore, it is warranted to briefly address potential negative health consequences that vegan diet-induced micronutrient deficiencies may have and strategies to mitigate them [6,227,228].

### Vitamin B12

Vitamin B12 (cobalamin) is a water-soluble vitamin essential for normal brain and nervous system functioning, DNA synthesis, and homocysteine metabolism and has indirect links to energy metabolism [[229], [230], [231], [232]]. Bioavailable vitamin B12 is stored in mammalian tissue and is subsequently almost exclusively available for human food intake in milk, dairy, eggs, and meat [233]. Moreover, naturally occurring bioactive forms of vitamin B12 are not found in plant foods (though some algae and fungi species do contain small quantities of vitamin B12 [234]), demonstrating the increased risk of deficiency in vegetarians and vegans [233,234].

The RDA for vitamin B12 is currently set at 2.4 μg/d for both male and female adults [11]. The EPIC-Oxford cohort study showed that 52% of vegans were vitamin B12 deficient (serum vitamin B12 < 118 pmol/L), and an additional 21% had low (but not deficient) serum vitamin B12 (118–150 pmol/L) [29], with similar data reproduced in United States and Germany large cohort studies [225, 235]. Though no analogous large-scale data are available specifically in vegan athletes, a recent study reported similar vitamin B12 plasma status across omnivorous, vegetarian and vegan athletes [236]. The disparity to the general population likely reflects higher overall energy intakes and the propensity for cobalamin supplementation in athletes (with nonsupplemented athletes reporting lower vitamin B12 levels irrespective of diet) [236]. Despite the low efficiency of cobalamin supplements [229,230], a significant positive association between supplementation and vitamin B12 status has been reported, indicating this as a viable strategy to offset low natural intakes [237]. Indeed, large sporting governing bodies (the International Olympic Committee, American College of Sports Medicine and International Society of Sports Nutrition) recommend vegan/vegetarian athletes to supplement with vitamin B12 or to consume vitamin B12 fortified foods (eg, fortified cereal or soy milk) to prevent cobalamin deficiency [20,238,239]. Current data therefore suggest that vegan athletes are at increased risk of vitamin B12 deficiency, and this can be prevented by cobalamin supplementation. However, the direct impact of deficiency on aspects of performance and adaptation are not clear, nor do any data show high(er) doses to be ergogenic.

### Iron

Iron is required for the formation of the oxygen-carrying proteins (hemoglobin and myoglobin) and mitochondrial enzymes, making it an essential component of oxygen transport and oxidative energy production [240]. Iron homeostasis is primarily regulated by hepcidin, a circulating peptide hormone secreted by the liver [241]. Once absorbed, the majority of iron is bound to ferritin and stored primarily in the liver, spleen, and bone marrow. As serum ferritin concentrations are highly correlated with total body iron stores, serum ferritin is used as a biomarker to estimate bodily iron stores [242]. Iron deficiency in athletes is typically classified into 3 levels of increasing severity [243]: iron stores below normal (serum ferritin <35 μg/L), iron-deficient nonanemia (serum ferritin <20 μg/L), and iron-deficient anemia (serum ferritin <12 μg/L).

The current RDA for iron, both for the general and athletic population, is 8 mg/d for males and 18 mg/d for females [244], with the higher female recommendations necessary to compensate for iron losses associated with menses [245] and lower hepcidin concentrations [246]. Iron deficiency is commonly observed in athletes, especially among endurance and female athletes, where the prevalence of iron depletion is up to 50% [[247], [248], [249], [250]]. Iron deficiency in athletes is caused by a combination of inadequate dietary iron intake from vegetarian or low energy diets, menstrual blood loss, or exercise-induced iron loss via sweating, hemolysis, and gastrointestinal bleeding [240,251]. Moreover, exercise-induced inflammation has been associated with increased hepcidin concentrations, which may result in decreased iron absorption and availability [252]. Low iron status is primarily characterized by tiredness, weakness, and shortness of breath [253] and has been associated with decreased endurance performance in a linear fashion [[254], [255], [256], [257]].

Dietary iron is available in 2 forms: heme and nonheme (heme refers to the organic compound attached to the iron molecule). The primary sources of heme iron are meat, poultry, and fish, whereas nonheme iron is derived from plant-based products such as cereals, pulses, fruits, and vegetables [258]. The quantity of nonheme iron is generally much higher than heme iron in a well-balanced diet. Unfortunately, nonheme iron is less bioavailable (2%–20%) than heme iron (15%–35%), due to the strong modulating effects of other food components on iron absorption [258], such as phytates and polyphenols commonly found in plant sources [258,259]. Therefore, although total iron is unlikely to be an issue in vegan athletes, their risk of deficiency is increased due to the lack of (bioavailable) heme iron in the diet. Given the athletes’ greater propensity for iron deficiency and the potential exacerbation of the issue by the vegan diet, iron supplementation may be worth consideration. Several studies have examined the ergogenic role of iron supplementation for athletes. Interestingly, iron supplementation in athletes without any form of iron deficiency does not improve indices of endurance performance [257]. In contrast, both oral iron supplementation and intravenous iron injections have reliably been reported to improve iron status and endurance performance in iron-deficient anemic athletes [255,257], with data on less severe nonanemia iron-deficient athletes more equivocal [254,260].

It is likely that a balanced diet rich in cereals, fruits, vegetables, and pulses should be sufficient for the male vegan athlete to maintain a sufficient iron status. Due to the higher recommended intake for females, achieving adequate iron intake through the diet might be more challenging, and additional oral iron supplementation may be considered [254,261]. Supplements have the added advantage that they typically contain ferrous salts, which are more bioavailable than dietary iron [262], with a possible drawback of increased likelihood for gastrointestinal disturbances [263]. For athletes deemed to require intravenous or intramuscular parenteral iron administration, this should be applied only under medical guidance (given risk for infection and/or toxic level doses [264]) as an effective strategy to rapidly restore iron status [257,265].

### Calcium

Calcium is a mineral that plays a key role in bone formation and, therefore, bone health. More than 99% of total body calcium is stored in bones and teeth. The remaining calcium is stored in muscle, the bloodstream, and in extracellular fluid where it is involved in the functioning of the muscular, cardiovascular, endocrine, and nervous system [266]. The primary dietary source of calcium is dairy, which accounts for approximately 70% of total calcium intake [267]. Other calcium-rich food sources are vegetables, including Chinese cabbage, kale, and soybean. Though individual foods differ, only ∼30% of dietary calcium is absorbed in healthy adults [267,268], and calcium-binding food components such as phytic acid, oxalic acid, and cellulose found in plant and whole-grain products lower calcium absorption further, highlighting the relevance of dietary calcium to the vegan athlete [267].

The current RDA for calcium intake in healthy adults aged 19 to 50 y is 1000 mg/d in the United States and 700 mg/d in the United Kingdom [267,269]. Data from the EPIC-Oxford cohort illustrate that average calcium intakes were 1042 mg/d and 988 mg/d in UK males and females, respectively [26]. However, categorized by diet, daily calcium intakes were significantly lower in both male (610 mg/d) and female (582 mg/d) vegans [26], suggesting deficiency in vegans would be prevalent. Indeed, this cohort had a significantly higher fracture risk compared with meat eaters, fish-eaters, and vegetarians [270]. In support, a recent systematic review and meta-analysis revealed that vegans had a lower bone mineral density than omnivores [271]. Collectively, these data provide convincing evidence for the vegan athlete to be at increased risk of calcium deficiency and associated adverse effects on bone health.

Calcium homeostasis does not seem to be influenced by exercise. Despite an acute decline in plasma calcium concentrations following a single bout of exercise [[272], [273], [274]], this appears transient given prolonged periods of training do not modulate plasma calcium levels [[275], [276], [277]], implying athletes per se are not at risk of disturbed calcium homeostasis. However, specific attention should be paid to female (vegan) athletes due to the phenomenon of the female athlete triad, a complex disorder that develops as a consequence of a dysfunctional interrelation between menstrual dysfunction, low energy availability, and decreased bone mineral density, that can have serious health implications [278,279]. Consequently, increased calcium intakes of up to 1500 mg/d have been proposed in amenorrhoeic athletes [280]. It is therefore advised that vegan athletes monitor their calcium status and consume calcium-fortified foods or calcium-rich food products. In cases where athletes are susceptible to calcium deficiency and are unable to correct using dietary strategies, calcium supplements are widely available in 2 forms, calcium bicarbonate (40% elemental calcium) and calcium citrate (20% elemental calcium), both of which have similar intestinal absorbability [267]. Although rare, hypercalcemia (serum calcium levels ≥10.5 mg/dL), most likely as a result of oversupplementation, is associated with renal dysfunction, kidney stone formation, and vascular and soft tissue calcification [267]. Therefore, upper limits have been set at 2500 mg/d for adults aged 19 to 50 y and 2000 mg/d for adults aged >50 y [267].

### Vitamin D

Vitamin D is a fat-soluble vitamin and exists as 2 biologically active isoforms: vitamin D2 (ergocalciferol) and vitamin D3 (cholecalciferol). The 2 forms differ in side-chain structure but behave identically with respect to metabolic function [267]. Vitamin D is obtained from dietary sources (both D2 and D3) and synthesized in the skin (D3 only) from 7-dehydrocholesterol following exposure to sunlight [281]. Cutaneously produced vitamin D accounts for approximately 80% of total vitamin D supply [282] but can vary based on a number of aspects including the time of day, season of the year, latitude, skin pigmentation, and age [283,284]. This can be particularly relevant to athletes where seasonal fluctuations in vitamin D status have been observed [285] as well as differences between indoor and outdoor sport athletes [286]. Dietary-derived vitamin D2 is typically found in plants, mushrooms, and yeast, whereas vitamin D3 is contained in animal-based foods such as meat, fatty fish, cheese, and egg yolk [281]. Dietary sources are naturally relatively low; in fact, most ingested vitamin D in modern (Western) diets is actually consumed within fortified foods. For example, fluid milk is fortified with vitamin D3 in several countries such as the United States [267]. Additionally, milk substitutes (eg, soy or almond milk), yogurts, and ready-to-eat breakfast cereal products are commonly fortified with vitamin D3 [267]. Further, vitamin D supplements containing either vitamin D2 or D3, which have similar bioavailability as dietary-derived vitamin D [287], have become more popular and are commonly consumed [267].

Serum 25(OH)D concentrations (a marker of endogenously produced and dietary-derived vitamin D2 and D3) are used to determine vitamin D status. Healthy serum 25(OH)D levels are considered ≥50 nmol/L [288], but estimates indicate that 20% to 80% of US, Canadian, and European adults have serum 25(OH)D levels below this [289]. Regarding dietary vitamin D intake, the RDA for people aged 1 to 70 y is 15 μg (600 IU) in the United States and 10 μg (400 IU) in the United Kingdom [267,290]. Dietary intake data (excluding supplementation) from the United Kingdom show that mean vitamin D intake per day in adult vegans was only 0.7 μg compared with an intake of 3.1 μg/d in omnivores [30]. Indeed, when controlling for season, year of blood collection, and age, this considerably lower dietary intake translates to serum 25(OH)D concentrations in meat eaters being considerably (∼20 nmol/L) higher than in vegans [30]. This disparity between omnivorous and vegan diets appears to be a reliable finding given the same has been observed in a large Danish cohort study [291]. When athlete populations are considered, a meta-analysis across 23 studies with 2312 athletes revealed that more than 50% had low serum 25(OH)D concentrations (ie, ≤50 nmol/L) [292], although dietary habits were not discerned. Collectively, therefore, data suggest vitamin D status is a concern for athletes, and likely more so in those adhering to vegan diets.

Consequences of (prolonged) stark vitamin D deficiency (serum 25(OH)D <30 nmol/L) can result in inadequate bone mineralization [293] and osteomalacia (a disorder in which bones are softened). In vitamin D deficient athletes, oral supplementation restores serum 25(OH)D concentrations [294,295] and, consequently, improves various aspects of muscle performance [296]. In addition to restoring deficient levels, data are also emerging to suggest that vitamin D supplementation aimed at achieving serum concentrations above the reference value may augment increases in skeletal muscle mass, function, decrease recovery time from exercise, and increase both power and force production in older adults [284,297,298]. However, to date, whether an ergogenic effect of vitamin D supplementation in athletes with healthy or elevated serum 25(OH)D levels is equivocal [239,292,294,299]. Worthy of note, a tolerable upper level of supplementary vitamin D intake has yet to be established. Although serum 25(OH)D levels >185 nmol/L are generally considered toxic, a supplemental vitamin D intake of up to 70,000 IU/wk in athletes is considered safe [300].

Taken together, the increased risk for vitamin D deficiency in the vegan athlete is likely obviated by pre-emptive supplementation, especially if serum monitoring is possible. This may be of most relevance for athletes with limited sunlight exposure (ie, indoor sports or living at high latitudes). Aside from specific (bioavailable) supplementation, other prophylactic strategies that can be adopted are to focus on vitamin D fortified food products.

### Summary, Conclusions and Future Directions

There is a continued trend for an increase in the prevalence of the vegan diet in the general population, especially in Western cultures. As more elite performers continue to adopt and publicize the vegan diet, it is likely that the popularity of the vegan diet will also grow among athletes. Therefore, it is critical to assess whether athletes are able to follow a vegan diet without compromising performance, adaptation or recovery. To date, there is little empirical evidence comparing vegan and omnivorous diets in athletes and their effect on measures of adaptation and performance, making it difficult to draw direct conclusions on the wholesale outcomes of adopting a vegan diet. Therefore, we must rely upon data documenting the dietary intake of vegans (primarily in the general population) to make inferences about how any shifts in energy/nutrient intake are likely to impact the athlete.

**FIGURE 1:**
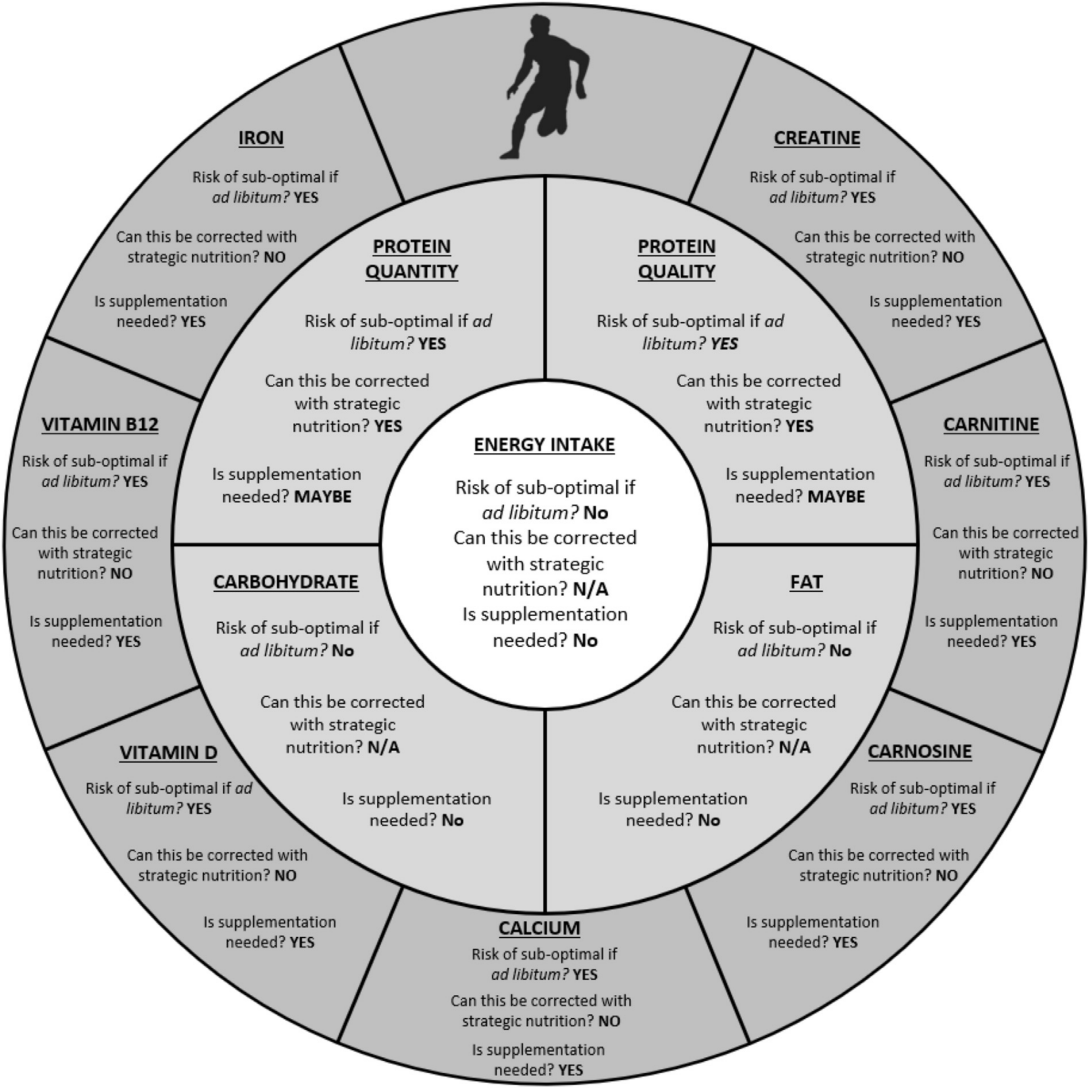
Schematic of the nutritional considerations of the vegan athlete. The graphic illustrates the key considerations when adopting a vegan diet with the relative importance (measured as impact on adaptation, performance, and recovery) increasing from outside to in. Each section follows the key theme of the review; that is, is there a risk of suboptimal nutrition when following an ad libitum vegan diet? Can this be made optimal through strategic nutritional planning alone? Is supplementation necessary to mitigate any deficiency?. N/A, not applicable.

Although there are no data to suggest an athletic benefit to adopting a vegan diet, it would also appear that one can follow a vegan diet without impairing performance or adaptation. The number of elite athletes following a vegan diet while training and competing at the top level is testimony to the fact that the vegan diet is capable of supporting elite athletes. However, such anecdotal reports are likely underpinned by careful planning and application beyond what is required from an omnivorous diet (Figure 1). Overall energy intake does not appear to be (majorly) compromised with a vegan diet; however, there is a shift in macronutrient composition. Although total carbohydrate and fat intake are not of concern, the athlete may lose some flexibility to manipulate macronutrient intake (ie, low carbohydrate diet) due to the high carbohydrate nature of plant-based sources. More consideration will need to be given to both the quantity and quality of the protein ingested, as it appears that below a certain (currently undetermined) threshold, both acute muscle protein turnover and chronic adaptation may be impaired with non–animal-derived sources/diets. Although meeting higher protein demands is achievable with a vegan diet, sufficient attention will need to be paid to ensure the athlete is consuming enough high-quality protein in the diet. Future research into non–animal-derived protein sources is warranted to identify optimal doses (per serving and per day), sources, and supplementation strategies to maximize phenotypic adaptation. The vegan diet is low or devoid of ergogenic compounds such as creatine, carnitine, and carnosine, which is also reflected in lower plasma and muscle concentrations. Although there are minimal data to suggest that these lower concentrations will impair adaptation and performance, supplementation with these popular ergogenic aids is worth considering for any (particularly vegan) athlete. Finally, athletes may be at particular risk of specific micronutrient deficiencies which may be exacerbated by the vegan diet. Therefore, athlete micronutrient status should be monitored, and any deficiencies corrected through diet (fortification) or supplementation if necessary.

## Acknowledgments

The authors’ responsibilities were as follows—SW: prepared the figures and tables and performed the literature searches; SW, AJM, IvdH, FBS, BTW: drafted the manuscript and all authors read and approved the final manuscript.

### Funding

FBS and BTW have received funding from Marlow Foods LTD relating to the content of this review.

### Author disclosures

AJM receives postdoctoral financial support, and FBS and BTW are currently receiving research grant funding from Marlow Foods LTD to perform research related to the content of this review. SW and IvdH are supported by studentships in collaboration with Marlow Foods LTD.

## Appendix A Supplementary data

Multimedia component1 Multimedia component1
