# Supplementary material for: Nutritional Considerations for the Vegan Athlete
Source: Adv Nutr. 2023 Apr 29;14(4):774–95. doi: 10.1016/j.advnut.2023.04.012 (PMC10334161; doi:10.1016/j.advnut.2023.04.012)
Supplement: Multimedia component1 [file mmc1.docx]

**Nutritional considerations for the vegan athlete.**

Sam West

**Supplementary material**

**Supplementary Table 1**. Example diets for an 82kg omnivore and vegan strength athlete.

|  | **Omnivore** |  | **Vegan** |
| --- | --- | --- | --- |
| **Breakfast** |  |  |  |
|  | Banana |  | Banana |
|  | Porridge oats |  | Porridge oats |
|  | Whole Milk |  | Soy Milk |
|  | Honey |  | Maple syrup |
|  | Orange Juice |  | Orange Juice |
|  | Whey Protein Isolate |  | Soy Protein Isolate |
| **Lunch** |  |  |  |
|  | Banana |  | Banana |
|  | Broccoli |  | Broccoli |
|  | Chicken |  | Quorn Vegan Pieces |
|  | White Rice |  | White Rice |
|  | Wholemeal Deli Rolls |  | White Bread |
|  | Roast Turkey Breast |  | Vegan Butter |
|  | Butter |  | Strawberry Jam |
| **Dinner** |  |  |  |
|  | Asparagus |  | Kidney Beans |
|  | Sirloin Steak |  | Carrots |
|  | Olive Oil |  | Celery |
|  | New potatoes |  | Garlic |
|  |  |  | Lentils |
|  |  |  | Chopped Tomatoes |
|  |  |  | Olive Oil |
|  |  |  | Red Onion |
|  |  |  | Red Pepper |
|  |  |  | Soya Mince |
|  |  |  | Vegetable Stock |
|  |  |  | White Rice |
| **Snacks** |  |  |  |
|  | 1 x Apple |  | 2 x Marmite Rice Cakes |
|  | 1 x Banana |  | 1 x Trek Bar flapjack |
|  | Doritos |  | 2 x Apple |
|  | Grapes |  | 2 x Banana |
|  | Malt Loaf |  | Grapes |
|  | Whey Protein Isolate |  | Soy Protein Isolate |
| **TOTAL** |  |  |  |
| Energy (kcal) | 3564 |  | 3552 |
| Carbohydrate (g) | 490 |  | 567 |
| Carbohydrate (g/kg) | 6.0 |  | 6.9 |
| Protein (g) | 200 |  | 183 |
| Protein (g/kg) | 2.4 |  | 2.2 |
| Fat (g) | 89 |  | 61 |

**Supplementary Table 2**. Example diets for a 78kg omnivore and vegan endurance athlete.

|  | **Omnivore** |  | **Vegan** |
| --- | --- | --- | --- |
| **Breakfast** |  |  |  |
|  | 1 x Banana |  | Banana |
|  | Wholemeal toast |  | Soya Milk |
|  | Jam |  | Porridge Oats |
|  | butter |  | Maple Syrup |
|  | Porridge Oats |  | Orange Juice |
|  | Semi skimmed milk |  |  |
|  | Orange Juice |  |  |
| **Lunch** |  |  |  |
|  | Whole meal deli rolls |  | White Pasta |
|  | Butter |  | Red Pepper |
|  | Ham |  | Broccoli |
|  | White Pasta |  | Dolmio Pasta Sauce |
|  | Red pepper |  | Banana |
|  | Dolmio Pasta Sauce |  |  |
|  | Chicken |  |  |
| **Dinner** |  |  |  |
|  | White Rice |  | White Rice |
|  | Onions |  | Chickpeas |
|  | Garlic |  | Broccoli |
|  | Cumin |  | Chilli Flakes |
|  | Chilli Powder |  | Coconut Cream |
|  | Tomato Puree |  | Garlic |
|  | Chopped Tomatoes |  | Ginger |
|  | Beef Mince |  | Red Lentils |
|  | Vegetable Oil |  | Onion |
|  | Oregano |  | Baby Spinach |
|  |  |  | Tomatoes |
|  |  |  | Yeast Extract |
|  |  |  | Tumeric |
|  |  |  | Olive Oil |
| **Snacks** |  |  |  |
|  | 2 x Apple |  | Alpro Yoghurt |
|  | 3 x Banana |  | Sports Energy Drink |
|  | 1 x Breakfast bar |  | 2 x Apple |
|  | Malt loaf |  | 2 x Banana |
|  | Sports Energy Drink |  | Grapes |
|  |  |  |  |
| **TOTAL** |  |  |  |
| Energy (kcal) | 4036 |  | 4040 |
| Carbohydrate (g) | 637 |  | 649 |
| Carbohydrate (g/kg) | 8.2 |  | 8.3 |
| Protein (g) | 149 |  | 136 |
| Protein (g/kg) | 1.9 |  | 1.7 |
| Fat (g) | 99 |  | 100 |
